# From Early-Life to Late-Life: Patterns and Determinants of Mental Health in Older Chinese Adults

**DOI:** 10.1093/geroni/igaf122.1445

**Published:** 2025-12-31

**Authors:** Qian Song, Michelle Putnam

## Abstract

Depressive symptoms are highly prevalent among Chinese older adults, with studies indicating that nearly a quarter experience such symptoms. By the 2010s, this figure had risen to nearly 40%, alongside a 13% prevalence of cognitive impairment. This symposium brings together five papers that explore the early- and late-life determinants of psychological and cognitive health in older Chinese adults. The research topics span childhood food insecurity, job loss, late-life living arrangements, social engagement, and end-of-life hospice care. Wuyi examines the long-term impact of childhood food insecurity on depression trajectories, considering the roles of hukou status and the timing of food insecurity. Dr. Song et al. analyze the effects of involuntary job loss on late-life depressive symptoms, focusing on the rural-urban hukou divide and work ownership differences. Dr. Zhang et al. assess how late-life living arrangement transitions impact depressive symptoms, particularly among rural and urban older adults. Zhang et al. explore the effects of reduced social engagement on cognitive function during the COVID-19 pandemic, emphasizing gender and urban-rural disparities. Lastly, Yi et al. investigate depression and anxiety symptom networks in Chinese hospice patients, comparing different symptom profiles. Together, these studies deepen our understanding of the social determinants of mental and cognitive health in older Chinese adults, offering insights for policy and intervention development. Chinese Gerontology Studies Interest Group Sponsored Symposium

